# Tuberculosis household accompaniment to improve the contact management cascade: A prospective cohort study

**DOI:** 10.1371/journal.pone.0217104

**Published:** 2019-05-17

**Authors:** Courtney M. Yuen, Ana K. Millones, Carmen C. Contreras, Leonid Lecca, Mercedes C. Becerra, Salmaan Keshavjee

**Affiliations:** 1 Division of Global Health Equity, Brigham and Women’s Hospital, Boston, Massachusetts, United States of America; 2 Department of Global Health and Social Medicine, Harvard Medical School, Boston, Massachusetts, United States of America; 3 Socios En Salud Sucursal Peru, Lima, Peru; 4 Partners In Health, Boston, Massachusetts, United States of America; South African Medical Research Council, SOUTH AFRICA

## Abstract

**Background:**

Appropriate management of people exposed in the home to tuberculosis is essential to prevent morbidity. These household contacts, particularly children, should receive preventive therapy to prevent them from falling ill. However, few people receive preventive therapy worldwide. We sought to determine whether a community-based accompaniment intervention could improve tuberculosis contact management.

**Methods:**

We conducted a prospective cohort study of household contacts of tuberculosis patients who initiated treatment during September 2015-June 2016 in Lima, Peru. Enrolled households received an intervention comprising home visits, transport vouchers, assistance coordinating evaluation procedures, and adherence support during preventive therapy. To evaluate the impact of the intervention, we conducted retrospective chart reviews of all patients initiating treatment during 6-month baseline and intervention periods.

**Results:**

We enrolled 314 household contacts of 109 index patients. Of these, 283 (90%) completed evaluation, and 4 (1%) were diagnosed with tuberculosis. Preventive therapy was prescribed for 35/38 (92%) contacts 0–19 years old who were eligible under Peruvian guidelines. Preventive therapy was also prescribed for 6/26 (23%) contacts with unknown eligibility due to lack of a tuberculin skin test (TST), and 20/69 (29%) who were ineligible either because of a negative TST result or exposure to a drug-resistant or extrapulmonary case. Of the 61 contacts who were prescribed preventive therapy, 57 (93%) initiated treatment, and 51 (91%) completed treatment. The proportion of contacts who completed evaluation increased from 42% during the baseline period to 71% during the evaluation period (risk ratio [RR] = 1.73, 95% confidence interval [95% CI]: 1.41–2.13). The proportion of contacts who initiated preventive therapy increased from 15% to 40% (RR = 2.45, 95% CI: 1.42–4.22).

**Conclusion:**

Accompaniment of TB patient households greatly improved the evaluation of household contacts for TB and increased the use of preventive therapy.

## Introduction

People living in the household of a tuberculosis (TB) patient are at high risk of getting infected with *Mycobacterium tuberculosis* and developing TB disease, especially young children and people with immune-compromising conditions [1]. In low- and middle-income countries, around 2–5% of household members of TB patients are typically diagnosed with TB disease, with children and people living with HIV at particularly high risk [1]. As a result, the evaluation of all household contacts of TB patients (via varied combinations of clinical evaluation, sputum testing, chest radiography, and tuberculin skin testing), are universal recommendations in national TB policies [2, 3]. The provision of preventive therapy to young child contacts is also a universal recommendation, and most guidelines recommend preventive therapy for contacts living with HIV [3]. However, during 2017, only 23% of children <5 years old who should have received preventive therapy are estimated to have received it [4]. Even fewer adult contacts are likely to have received preventive therapy given that HIV-negative adults are not eligible for treatment under most national policies [3].

Ensuring appropriate management of household contacts of TB patients is logistically challenging. The cascade of care for contact management presents many opportunities for drop-off: contacts may not be identified, evaluation procedures may not be completed, and preventive therapy may not be prescribed, initiated, or completed [5]. Certain barriers have been consistently reported from diverse settings [5], including contacts facing difficulties traveling to health facilities for evaluation [6, 7], knowledge gaps among healthcare workers and patients [8, 9], and low acceptability of taking medications among individuals who do not feel sick [6, 9]. In addition, treatment completion is poor with the 6-month isoniazid regimen that is recommended in most high-burden countries [5], which have yet to adopt shorter rifamycin-based regimens that are associated with better completion [10]. Finally, many countries lack clear guidelines on the management of contacts of patients with drug-resistant TB, which is resistant to the drug classes that are most commonly used for preventive therapy [3].

Comprehensive strategies, such as those espoused by global TB elimination programs like the Zero TB Initiative [11], require expanding the use of preventive therapy beyond young children and people living with HIV. The importance of expanding the use of preventive therapy is supported by published TB elimination models [12, 13] and recently updated recommendations from the World Health Organization [14]. To effectively expand the use of preventive therapy, implementation barriers that have previously been identified will have to be surmounted.

Peru is a middle-income country with an estimated TB incidence of 116 cases per 100,000 population, which is over ten times higher than the target of 10 cases per 100,000 that defines low incidence [4]. Peru’s national TB policy indicates both baseline and follow-up evaluations for contacts of all TB patients, and recommends preventive therapy for contacts up to 19 years old [15]. These policies provide a more intensive form of TB contact management than is recommended in many settings, which require only a baseline evaluation and limit preventive therapy to young children [3]. However, significant barriers to implementing Peru’s policies have been documented. For instance, TB patients and their families often lack resources to travel to health facilities [16, 17], access to chest radiography contact evaluation is limited [17], and despite the large number of clinical trials showing the benefits of preventive therapy [18, 19], some providers feel ambivalence towards its use [20].

Effective strategies are needed for improving contact management within the context of the existing health system. In Peru, a strategy of conditional cash transfers in combination with community-based support groups has been shown to increase the proportion of contacts who receive preventive therapy [21, 22]. We conducted a prospective cohort study to evaluate a different intervention approach that used individualized accompaniment of TB patient households to improve contact management.

## Methods

### Study setting and population

Carabayllo is a district in northern Lima, Peru with a TB case notification rate around 100 per 100,000 population. Its population is served by nine public primary-level health facilities. According to Peru’s national TB policy, contacts of all TB patients should be identified, registered, and evaluated at the health facility for TB [15]. After ruling out TB disease, contacts of patients with pulmonary drug-sensitive TB should be given isoniazid preventive therapy (IPT) for 6 months if they are either (a) under 5 years old or (b) between 5–19 years old and have a positive tuberculin skin test (TST) result (≥10 mm induration) regardless of HIV status. All contacts of drug-sensitive TB patients should undergo another TB disease evaluation when the index patient changes to the continuation phase of treatment and when the index patient finishes treatment; contacts of drug-resistant TB patients should be re-evaluated every 3 months until the index patient completes treatment. For both baseline and follow-up evaluations, the use of clinical evaluation, bacteriologic testing, and radiography are indicated in the guidelines, but the exact algorithm is not specified.

During 2015–2016, the non-governmental organization Socios En Salud collaborated with the Ministry of Health and the Municipality of Carabayllo to implement the TB Cero initiative (part of the global Zero TB Initiative), which aimed to avert TB deaths through active case-finding, comprehensive patient support, and improved use of preventive therapy [16]. Patient support was delivered programmatically and has been previously described [16, 23]. The accompaniment intervention to improve contact management was implemented as a prospective cohort study nested within the TB Cero activities. For this study, household contacts were defined as people who were living in the same residence as the index patient at the time of enrollment.

### Intervention

A community-based accompaniment team of trained community health workers and nurse technicians from Socios En Salud implemented the intervention. They worked with TB-affected households enrolled in the study and with the health center staff providing care to increase the understanding of the importance of contact management and to reduce barriers to completing the recommended procedures. Clinical care was managed per routine practice by Ministry of Health clinicians in primary care facilities.

The initial phase of the accompaniment intervention comprised home visits to encourage household members to complete evaluation, transport vouchers to help them get to and from health facilities (less than $5 round trip), and assistance coordinating appointments for TST, chest radiography, and pediatric TB specialist consultations when indicated by the primary care physician. The follow-up phase of accompaniment comprised two home visits to encourage household members to go to health facilities for a repeat TB evaluation; per Peruvian guidelines, these visits occurred after the index patient entered the continuation phase and after the index patient completed treatment for drug-sensitive TB, and at 3 and 6 months for drug-resistant TB. During these visits, a symptom screen was administered so that symptomatic individuals could be prioritized for evaluation; transport vouchers were offered as necessary, based on a questionnaire for assessing unmet needs [16] and the judgment of the study staff.

For contacts who initiated IPT, more intensive support was offered, including home-based supervised IPT administration with adherence counseling by a community health worker, and monthly home visits by a nurse technician to monitor adverse events during IPT. For contacts who consented to home-based supervised IPT administration, all medications were brought to the home on a daily basis, with supervised administration either 6 or 7 days per week. If supervision took place Monday–Saturday, then the community health worker left the Sunday dose at the home for self-administration. Adverse event monitoring was conducted in the home by study staff, who administered a standardized questionnaire about the occurrence of adverse events, followed by referral for clinical evaluation for any event that was concerning.

### Intervention enrollment and data collection

We recruited adult (at least 18 years old) newly diagnosed index patients, including all types of TB and resistance patterns. We asked index patients to give written informed consent to allow us to recruit their household members. The latter were then recruited and asked to provide written informed consent; for minors, guardians were asked for consent and children at least 8 years old were also asked for assent. All TB patients who initiated treatment between September 2015 and June 2016 in one of the nine public health facilities of Carabayllo district and their household members who lived in Carabayllo district were eligible for recruitment. Eligibility was briefly expanded to include patients initiating treatment in three similar health facilities in the neighboring Comas district as part of a programmatic expansion that was stopped due to lack of funding.

Enrolled participants agreed to receive household visits from the community accompaniment team and to allow the outcomes of all evaluation procedures to be collected from their clinical records. In addition, data on symptom screening and adverse events during IPT were collected through direct interaction with participants. Enrolled participants could opt out of receiving any of the support offered without withdrawing from the study.

### Evaluation of implementation

We evaluated the implementation of the accompaniment intervention among enrolled contacts by assessing the proportion of enrolled contacts who completed key recommended procedures, including baseline diagnostic evaluation, follow-up symptom screens, and follow-up evaluations at a health facility. At both baseline and follow-ups, we defined participants as having been evaluated if a TB evaluation result was recorded at the health facility. We assessed the proportion of participants diagnosed with TB, the cascade of care for children eligible for IPT, and the proportion of children reporting adverse events during IPT.

### Evaluation of impact

We wished to assess whether offering community-based accompaniment resulted in better contact management across the entire target population, not only among those who chose to enroll. Therefore, our impact evaluation strategy was to conduct a pre/post comparison that included contacts of all patients initiating treatment during the designated comparison periods, regardless of whether or not they enrolled in the study. In this way, we sought to remove the effect of selection bias in enrollment. Furthermore, to avoid potential bias caused by better record-keeping in the context of a study, we did not use any study documents in the impact evaluation, but rather relied on data routinely collected by health facilities.

For the baseline period in this impact evaluation, we chose a 6-month period before study enrollment started (March–August, 2015); contacts of TB patients initiating treatment during this period were not offered any intervention. For the intervention period of the impact evaluation, we chose a 6-month period during which the intervention was actively enrolling (October, 2015–March, 2016. We conducted retrospective chart reviews of all patients initiating treatment in the nine health facilities of Carabayllo during these baseline and intervention periods. We extracted information from standardized contact management forms in patient charts that record the identification of contacts, the results of TB evaluation procedures, the prescription of IPT, and IPT delivery based on pharmacy records. These forms are routinely filled out by health facility staff; Socios En Salud intervention staff played no role in completing these forms during the intervention period.

We assessed the contact management cascade of care by calculating the percentage of contacts who completed each management step out of those who were eligible to complete it [5, 24]. Statistical testing was performed by calculating risk ratios using a modified Poisson regression with generalize estimating equations to account for household-level clustering. We visually illustrated the under-5 child contact management cascade by multiplying the percentages of eligible children who completed each step to depict cumulative cascade losses at each step. Statistical analysis was performed in SAS 9.4 (SAS Institute, Cary, NC).

### Ethical approval

The prospective cohort study was approved by the Institutional Review Board of Harvard Medical School and the Institutional Committee for Research Ethics of the National Institute of Health in Peru. The retrospective impact evaluation was approved by the Institutional Review Boards of Partners Healthcare and Cayetano Heredia University.

## Results

### Contact evaluation and IPT prescription

**Fig 1:**
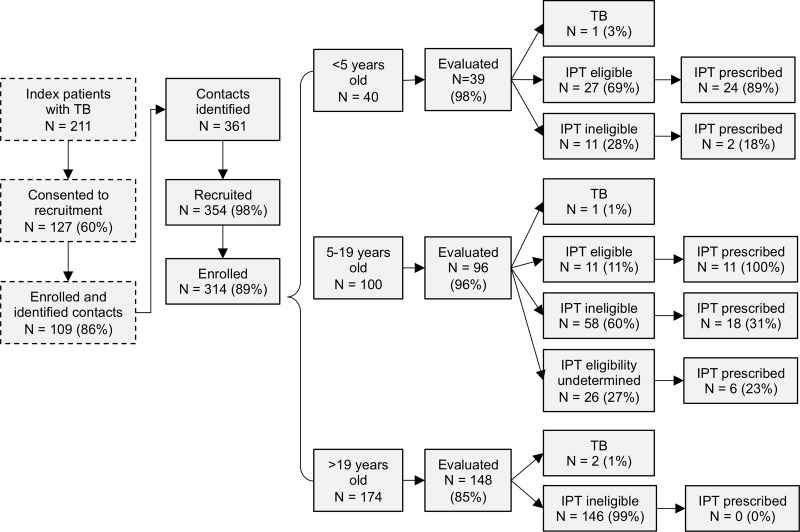
Participant enrollment and baseline evaluation results for contacts (N = 314). Contacts were eligible for isoniazid preventive therapy (IPT) if they were exposed to drug-sensitive pulmonary TB and either <5 years old or 5–19 years old with a positive tuberculin skin test (TST) result. Contacts exposed to drug-resistant or extrapulmonary TB were ineligible for IPT. Contacts 5–19 years old without a TST were categorized as having undetermined IPT eligibility.

During the intervention period, 211 index patients were diagnosed with TB in participating health facilities. Of these, 127 (60%) consented to recruitment for the study, of whom 109 (86%) reported household contacts (Fig 1). Of consenting index patients with household contacts, 74 (68%) had pulmonary drug-sensitive TB, 20 (18%) had extrapulmonary TB, and 15 (14%) had multidrug-resistant TB. These index patients identified a total of 361 household contacts, with a median of 3 (range 1–11) contacts per index patient. Of the identified household contacts, 354 (98%) were approached for recruitment into the study, 314 (89%) of whom were enrolled.

**Fig 2:**
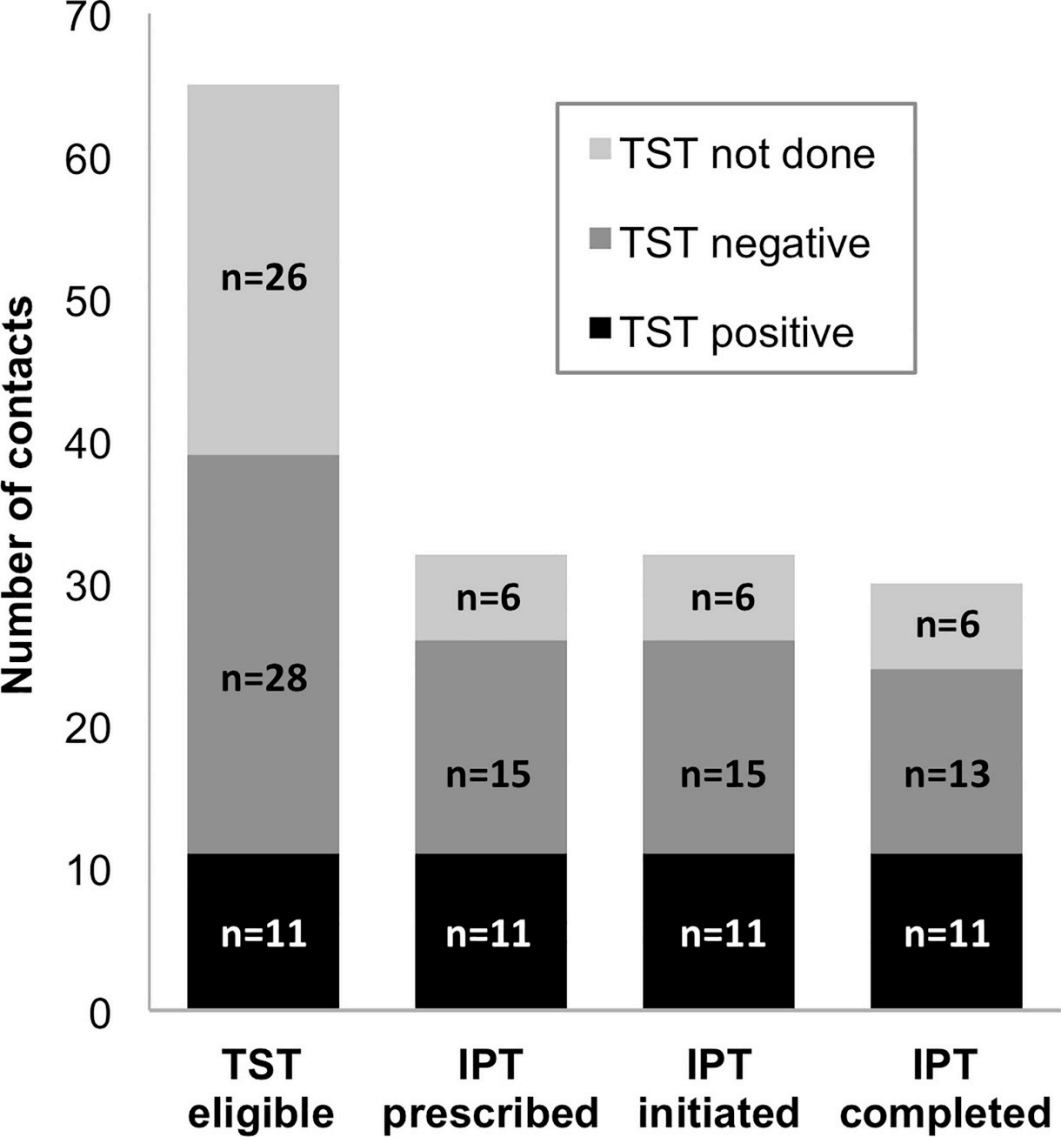
Isoniazid preventive therapy (IPT) cascade for contacts 5–19 years old exposed to drug-sensitive pulmonary TB, by tuberculin skin test (TST) status (N = 65).

In total, 283 (90%) enrolled contacts completed a TB evaluation, and 4 (1%) were diagnosed with TB, including one child under 5 years old (Fig 1). Of the 27 evaluated children under 5 years old who were eligible for IPT, 24 (89%) had IPT prescribed. Among the 96 evaluated contacts between 5 and 19 years old, 65 (68%) should have received a TST to determine their eligibility for IPT based on being contacts of patients with pulmonary drug-sensitive TB and having had TB ruled out; of these, 39 (60%) had a TST performed (Fig 2). IPT was prescribed for all 11 (100%) contacts with a positive TST result, 15 (54%) of those with a negative TST result, and 6 (23%) of those for whom TST was not performed. In total, across both age groups, IPT was prescribed for 61 contacts: 35/38 (92%) who were eligible for IPT under Peruvian guidelines, 6/26 (23%) with unknown eligibility due to lack of a TST, 2/24 (8%) who were ineligible for IPT based on being contacts of a drug-resistant index case, 3/17 (18%) who were ineligible for IPT based on being contacts of extrapulmonary index cases, and 15/28 (54%) who were ineligible for IPT based on having a negative TST result.

### IPT completion and adverse events

Of the 61 contacts prescribed IPT, 57 (93%) initiated IPT. Of these, 43 (75%) had caregivers accept home-based supervised administration of IPT, and 48 (84%) had caregivers accept monthly visits for adverse event monitoring. In total, 51 (91%) of the contacts who initiated IPT completed it; 3 (5%) moved out of the intervention district so treatment completion could not be determined, 1 (2%) had IPT suspended by the managing physician, and 1 (2%) stopped IPT independently.

Of the 48 contacts who received monthly adverse event monitoring visits, 46 (96%) received 5 or 6 visits. A total of 16 adverse events were reported for 10 (21%) contacts, most of which occurred only once and self-resolved (Table 1). No contacts experienced the same type of adverse event during multiple months. Skin rash was the most commonly reported adverse event. There were no reports of adverse events associated with hepatotoxicity (i.e. dark urine or yellowing of the eyes or skin) or neuropathy (i.e. numbness or tingling in the fingers or toes). Adverse events were reported more frequently in earlier visits (Cochran-Armitage trend test exact p = 0.0275). Only one child had IPT suspended because of adverse events; this occurred for a child who experienced a skin rash during the first month of IPT.

**Table 1:** Adverse events reported among child and adolescent contacts receiving isoniazid preventive therapy (N = 48).

Adverse event	Contacts experiencing adverse event once during the month, n (%)	Contacts experiencing adverse event >1 time during the month, n (%)
Nausea	1 (2)	1 (2)
Vomiting	0 (0)	0 (0)
Loss of appetite	1 (2)	0 (0)
Stomach ache	0 (0)	0 (0)
Insomnia	1 (2)	1 (2)
Dark urine	0 (0)	0 (0)
Yellowing of the eyes or skin	0 (0)	0 (0)
Skin rash	5 (10)	0 (0)
Numbness or tingling in fingers or toes	0 (0)	0 (0)
Fatigue or malaise	2 (4)	0 (0)
Other	2 (4)*	0 (0)

*1 report of headache, 1 report of dry lips

### Follow-up

**Fig 3:**
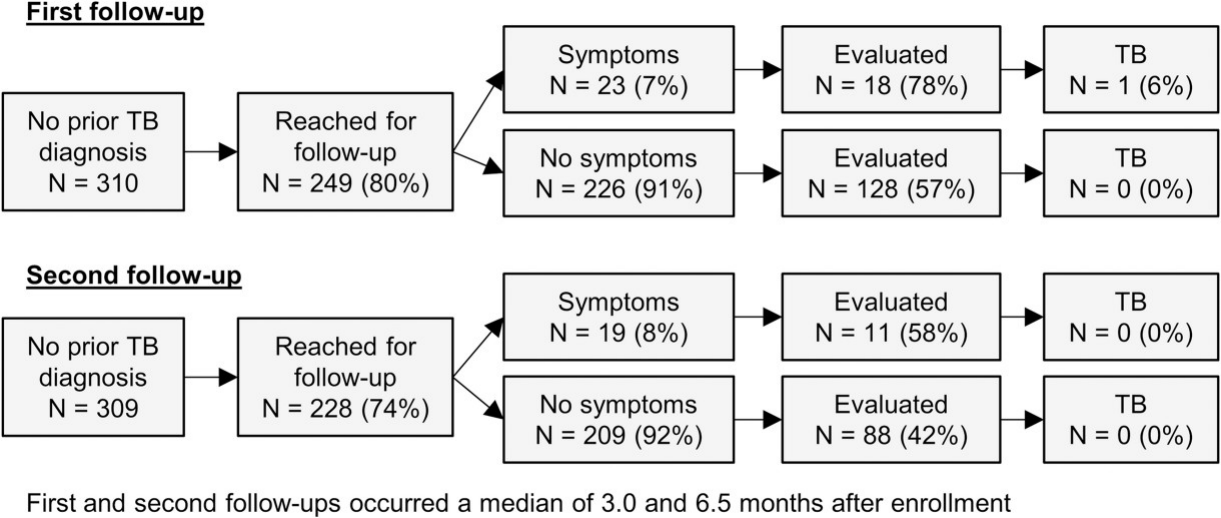
Contacts who were reached for symptom screening and who were evaluated at health facilities as part of first and second follow-up.

Of the 310 contacts who had TB disease ruled out during the baseline evaluation, 249 (80%) were reached for the first follow-up, which occurred a median of 3.0 (interquartile range [IQR] 2.5–3.8) months after enrollment. Of these, 146 (47%) were evaluated at a health center, and one was diagnosed with TB disease (Fig 3). The contact diagnosed with TB disease was an 18-year-old who had not received IPT because the index patient had MDR-TB. Of the 309 contacts not known to have TB disease after the first follow-up, 228 (74%) were reached for the second follow-up, which occurred a median of 6.5 (IQR 6.2–7.1) months after enrollment. Of these, 99 (32%) were evaluated at a health center, and none were diagnosed with TB disease. No contacts who were reached for follow-up reported having been diagnosed with TB disease during the time between follow-ups.

### Impact of accompaniment intervention on contact management in target population

For the evaluation of impact at the level of the target population, we reviewed charts from 145 patients who initiated TB treatment during the baseline evaluation period and 134 who initiated TB treatment during the intervention evaluation period. A total of 202 (42%) of 483 contacts identified in the baseline period and 320 (71%) of 450 contacts identified in the intervention period were evaluated clinically or bacteriologically, representing a significant increase (risk ratio [RR] = 1.73, 95% confidence interval [95% CI]: 1.41–2.13). Among contacts under 20 years old who were contacts of patients with drug-sensitive pulmonary TB, the proportion who initiated IPT increased from 16% to 40% (RR = 2.45, 95% CI: 1.42–4.22); a comparison among only those eligible for IPT was not possible given the lack of TST results to determine eligibility in contacts 5 to 19 years old. Among those who initiated IPT (N = 22 in the baseline period and N = 54 in the intervention period), the proportion completing IPT increased from 68% to 81%, but the difference was not statistically significant (RR = 1.17, 95% CI: 0.83–1.67).

**Fig 4:**
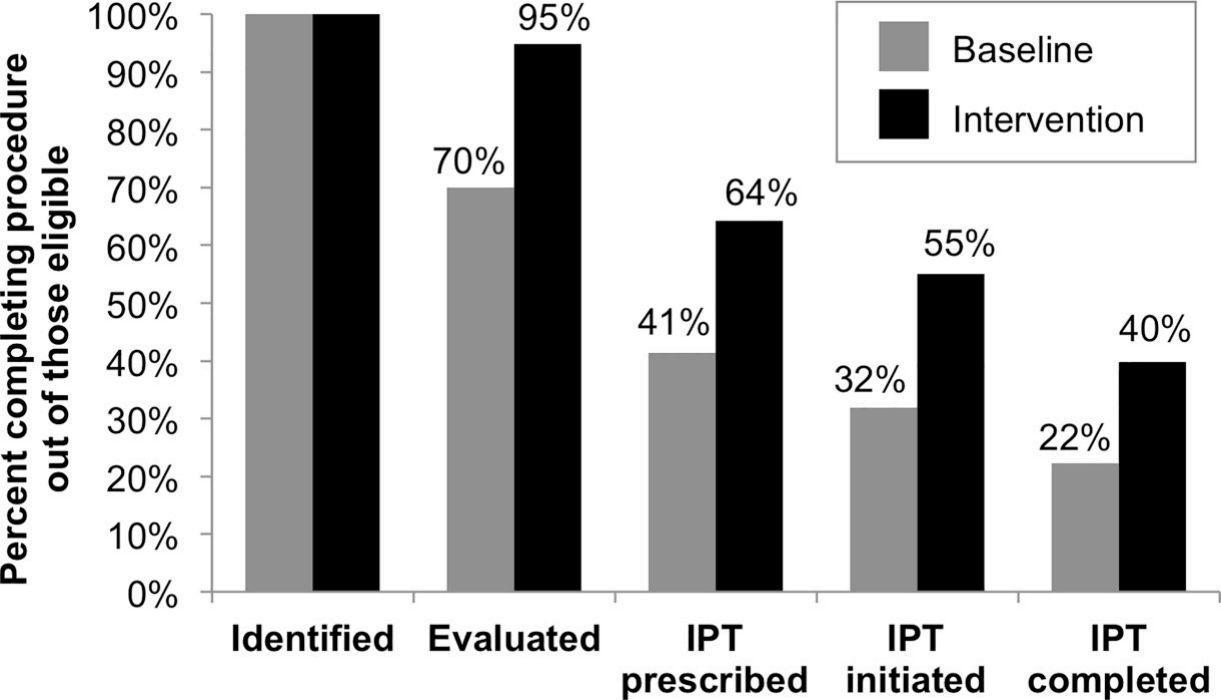
Evaluation of contact management cascade for child contacts <5 years old during baseline (N = 40) and intervention periods (N = 39). Cumulative percentages of contacts progressing through each cascade step are shown.

Among child contacts under 5 years old (40 in the baseline period and 39 in the intervention period), the percentage evaluated increased significantly from 70% during the baseline period to 90% during the intervention period (RR = 1.35, 95% CI: 1.07–1.71; Fig 4). Completion of other steps in the young child contact management cascade also improved, but the differences were not statistically significant. The largest gap in the cascade was in IPT prescription, with only 59% and 68% of eligible children being prescribed IPT in the baseline and intervention periods, respectively.

## Discussion

We found that accompaniment of TB patient households greatly improved the evaluation of household contacts for TB and increased the use of preventive therapy. Although the accompaniment intervention did not reach all TB households in the area, the high proportion of evaluated contacts among enrolled households resulted in a substantial increase in the evaluation of the TB contact population as a whole. However, because the intervention focused on supporting TB households, it was unable to address barriers within the healthcare system that resulted in sub-optimal prescription of IPT to children and adolescents. Finally, we found that even with active, community-based follow-up, re-evaluation of all contacts after 3 and 6 months, as recommended by Peru’s national policy, is difficult to implement.

For contacts to complete screening and evaluation procedures, it is necessary that they understand and accept the importance of these procedures, and that they have the means to complete them. Interventions to improve awareness of the importance of contact evaluation (e.g. through household visits or education of the index patient) may meet with limited success in improving contact management if barriers to accessing the necessary services remain prohibitive [7, 25]. Our intervention addressed education by developing trusting relationships between community health workers and families of TB patients and addressed access by having these community health workers help to coordinate healthcare appointments and transportation. In contrast to this individualized accompaniment approach, another successful intervention carried out in a different district of Lima used both household visits and community meetings to deliver education and provided conditional cash transfers to reduce access barriers [21, 26]. Thus, while there are multiple ways to improve contact evaluation within the context of the existing health system, it is critical to address the barriers in accessing services. Furthermore, while both transport vouchers and conditional cash transfers require resources, these costs must be weighed against cost of treating TB cases that could have been averted through effective delivery of preventive therapy.

Our experience shows that getting contacts to health facilities is necessary but not sufficient for ensuring appropriate use of preventive therapy, as around one third of eligible children <5 years old were not prescribed IPT during the intervention period. We found that primary care physicians were sometimes reluctant to prescribe IPT because they lacked confidence in ruling out TB disease, an observation consistent with documented challenges in pediatric TB diagnosis at the primary care level in Peru [17]. Primary care physicians referred child contacts to a specialist at the National Institute for Child Health; however, because parents faced difficulties in securing appointments and bringing the necessary documentation (including a chest radiograph), many of these referrals were not completed, resulting in a lack of IPT prescription. The higher proportion of children prescribed IPT among those enrolled in the study may have been attributable in part to the intervention’s assistance with coordinating the specialist visits, as primary care physicians were more comfortable prescribing IPT after an experienced pediatrician had ruled out TB disease. This suggests that provider-side interventions to improve the capacity for TB diagnosis at the primary care level are needed.

We also observed that TST results were not being used as expected for guiding prescription of IPT. Notably, among contacts 5–19 years old, IPT was prescribed more frequently for those with a negative TST result compared to those in whom a TST had not been performed. While we did not collect systematic data on the reasons for physicians’ decisions to prescribe or not prescribe IPT, one possibility is that some saw the negative TST result only as an effective way to rule out TB rather than a means for determining who would benefit most from preventive therapy. Thus, more work is needed to understand provider-side barriers to appropriate use of preventive therapy, and training may be necessary to help providers use infection testing appropriately to guide clinical decisions.

We found it challenging to ensure that contacts undergo the follow-up evaluations at 3 and 6 months that are required by Peru’s national policy. This policy is evidence-based given that during the first year of follow-up, contacts of TB patients have over 10 times the risk of developing TB disease as the general population [1]. However, we found that while it was possible to reach household contacts to perform a symptom screen, convincing them to spend the time and resources be evaluated at a health facility was difficult, particularly if they did not have any symptoms. Given the low sensitivity of self-reported symptoms for detecting TB disease [27], this is problematic. These challenges have implications outside Peru’s local situation, as other countries are starting to include long-term monitoring for patient and contacts in their treatment recommendations; notably, India, the country with the most TB patients in the world, has recommended 2 years of follow-up of TB patients after treatment [28]. To increase the completion of follow-up evaluations, strategies such as conditional monetary incentives [29] and increasing health system capacity to expand availability of appointments and reduce wait times [30] should be considered.

Our study had several limitations. First, because receipt of the intervention was voluntary, self-selection by households means that the high rates of procedure completion observed among the intervention households may not be generalizable. However, our results still suggest a benefit to offering the intervention since our impact evaluation showed improvement in contact evaluation among all TB households. Second, we did not record information on the implementation of individual support activities within the accompaniment intervention, so we could not assess the contributions of different components of the intervention to improved contact management. Thirdly, for all children not receiving supervised IPT as part of the intervention, we assumed IPT completion based on a record that medications were given by health center staff to caregivers; this is likely to have overestimated IPT completion. Because this overestimation would have occurred more in the baseline period, it would bias our impact evaluation toward not detecting an improvement in IPT completion even if one truly existed. Finally, our intervention only offered IPT to contacts <20 years old per Peruvian guidelines; thus we were not able to deliver IPT to the entire family as a unit–an approach that may have facilitated greater group participation–and left untreated an important reservoir for future household and community transmission of TB.

In conclusion, community-based accompaniment of TB households can improve the management of household contacts and increase the use of preventive therapy. Reducing patient barriers to evaluation is a key strategy for ensuring access to preventive therapy, but provider-side barriers must also be addressed. These lessons may also be applied for improving the testing and treatment of TB infection in other high-risk populations.
